# Effectiveness of NCD-Related Fiscal Policies: Evidence from the Pacific

**DOI:** 10.3390/nu15214669

**Published:** 2023-11-03

**Authors:** Shazna M. Buksh, Annie Crookes, John B. F. de Wit

**Affiliations:** 1 School of Law and Social Sciences, The University of the South Pacific, Suva 1168, Fiji; s.m.buksh@uu.nl (S.M.B.); annie.crookes@usp.ac.fj (A.C.); 2 Translational Health Research Institute, Western Sydney University, Penrith, NSW 2751, Australia; 3 Department of Interdisciplinary Social Science, Faculty of Social and Behavioural Science, Utrecht University, 3584 CS Utrecht, The Netherlands

**Keywords:** Pacific Island Countries, fiscal policy, obesity, non-communicable diseases, sugar-sweetened beverages, snacks, fruits and vegetables, price food choice, household access, unhealthy eating

## Abstract

Obesity in Pacific Island countries (PICs) has hit crisis levels, and the consequent high non-communicable disease (NCD) burden is devastating for their developing economies. Nutrition transitions from traditional, plant and seafood diets to a dependence on processed foods are at the core of the obesity and NCD epidemic in PICs. Fiscal policies are widely promoted as an effective mechanism to reduce consumption of unhealthy foods and increase consumption of fruits and vegetables. However, there are little data to evaluate the effectiveness of these policies as rates of NCDs and obesity in PICs continue to rise. This study used an online survey to recruit 4116 adults from six PICs: Fiji, Kiribati, Samoa, Solomon Islands, Tonga and Vanuatu. The study measured the consumption of and household access to sugar-sweetened beverages, ultra-processed packaged snacks, fruits and vegetables and attitudes on food prices and unhealthy eating. The study also assessed the relationship between consumption of these foods and drink and (1) household access, (2) price as a food choice motive, and (3) unhealthy eating attitudes. This study provides novel data on food-related behaviours in PICs, offers insights into the potential impact of NCD-related fiscal policies on food consumption and identifies other variables of interest.

## 1. Introduction

Obesity in Pacific Island countries (PICs) has hit crisis levels: twelve of the 15 countries that make up the PICs rank in the top 20 most obese countries in the world [1,2,3]. PICs also have some of the highest rates of non-communicable diseases (NCDs) and premature deaths due to NCDs in the world [3]. The health, social and economic impacts of the obesity and NCD burden are devastating for the struggling economies of these low- and middle-income island nations [2]. 

The causes of obesity are complex and comprise overlapping and interrelated factors [4]. Nevertheless, there is substantial agreement that nutrition transitions from traditional plant and seafood-based diets to diets high in processed, imported foods are at the core of the obesity epidemic in PICs. Imported foods, predominantly food products high in unhealthy meat, salt, sugar and unhealthy fats, now constitute 60–100% of the food supply across the region [5,6,7,8,9,10]. Comparisons of data between national nutrition surveys undertaken in the early 2000s and 8–11 year follow-up surveys indicate that fruit and vegetable consumption in PICs is low [11,12] and decreasing in some PICs, including Tonga, Tokelau, Solomon Islands and Nauru [12]. Consumption of sugar-sweetened beverages (SSBs) for adults was measured in only four PICs, and in three of these countries (i.e., Kiribati, Nauru and Tokelau), participants on average consume the equivalent of approximately four cups of SSBs daily [12]. Salt intake in PICs was also not consistently measured in national nutrition surveys. However, urine analyses of adult Tokelauans indicated twice the WHO recommended intake, with an average consumption of 10.1 g of salt per day [12]. In sum, although the available data are old and sporadic, they paint an overall bleak picture of Pacifica diets. 

To combat the high consumption of imported, processed foods and the low consumption of fruits and vegetables, WHO’s Global Action Plan on NCDs [13] includes strong recommendations regarding the introduction of fiscal policies to promote changes in consumer behaviour. Common fiscal tools include import tariffs, excise duties, and value added tax (VAT), with product-specific taxes tied to value, volume or nutrition thresholds [10]. While taxes may be levied at different points in the food system, the assumption is that these will result in higher product costs to the consumer, leading to two interrelated behaviour changes in the population: a significant reduction in the purchase and consumption of unhealthy and processed foods, and a significant increase in the purchase and consumption of healthy alternatives (e.g., water, unsweetened beverages, fresh fruits and vegetables). There is strong evidence that fiscal strategies will contribute towards both of these behaviour changes e.g., [14,15,16,17]. For example, modelling exercises have indicated fiscal policies which reduce the cost of fruit and vegetables by 21% and 13%, respectively, can increase consumption in a population by 10% (UK model) [18], and the WHO [19] reports a price reduction of even 10% should positively impact consumption. Furthermore, one modelling exercise undertaken on Solomon Islands suggested that a 40% tax on SSBs would directly reduce type 2 diabetes in the population by up to 24,000 cases, and any taxation policy raising consumer costs by 20% or more would significantly reduce consumption of these products [20]. Given these potential beneficial effects, fiscal policies have been a key consideration at national and regional PIC discussions of NCD-related interventions over the last two decades [5,21].

Specific taxes affecting soft drinks and SSBs have been introduced in almost all PICs, except Solomon Islands and Palau, where taxation on SSBs is the same as that on imported water [22], and Tokelau, which has moved towards a complete ban of SSBs. These policies have also evolved over time, with some PICs (e.g., Cook Islands and Kiribati) shifting from import tariffs to the more effective excise duties and some (e.g., Fiji, Nauru, Niue and Vanuatu) adopting increasing tax levies over time. At the time of this publication, SSB-specific import tariffs exist in Nauru and Niue; excise duties are implemented in Cook Islands, Samoa, Tonga and Kiribati; and both import and excise duties are in place in Fiji, French Polynesia, New Caledonia, Tuvalu and Vanuatu [22]. 

One explanation for changes in the implementation of SSB-related policies might be the challenges in maintaining political support: A major challenge for PICs as low- and middle-income countries is the need to balance health-related policies against the priority for economic development and trade [21,23,24,25,26,27]. For instance, countries like Fiji have experienced overt challenges to implementing fiscal policies from local manufacturers who are important economic players in these countries [24,25,26]. This may be one of the reasons that Fiji has lower taxes on locally produced SSBs than imported SSBs. Furthermore, the implementation of a tax on SSBs involves multiple decisions on when, how much and through what mechanisms tax will be levied, requiring negotiations with multiple stakeholders across government sectors with competing priorities [20,25]. It is likely that PICs lack the capacity to coordinate the required complex negotiations leading to sustainable polices and strong enforcement systems [20,24]. 

Progress reviews also indicate several weaknesses in the technical planning and design of fiscal policies in PICs that prevent them from being optimized for modifying consumer behavior [22,25]. Firstly, import tariffs on specific products may not lead to a reduction in consumption. For example, a case study of fruit and vegetable fiscal policies and consumption in Fiji found that while policies had reduced costs by up to 10% (2010–2014), this had not resulted in any change in consumer prices [14]. Similarly, the introduction of a 30% “sugar levy” in Nauru in 2007 was confounded by retailers importing cheaper products, resulting in no change in SSB costs to consumers [23]. Secondly, taxation policies that have a narrow focus may not adequately reduce unhealthy consumer choices. For example, the SSB tax policies in several PICs initially only targeted soft drink-beverages without including sugar-sweetened fruit juices, pure fruit juices with high natural sugar content, or sugar flavoured milk products [10]. Thirdly, excise duties tied to value or volume thresholds are common [22], and these could restrict local manufacturing and retail businesses, making them politically tenuous. In contrast, an excise duty with nutrient-specified criteria (as implemented in Cook Islands) allows and encourages re-formulation of products as a compromise that meets both health and industry priorities [22,25]. Lastly, the focus of NCD-related fiscal policies in PICs is almost exclusively on reducing unhealthy food consumption, without equivalent policies to incentivize consumption of healthier alternatives and fruits and vegetables [22]. A notable exception is the 2010 Fiji NCD action plan, which coupled specific import taxes on unhealthy food products, such as palm oil, with a reduction and removal of import duties on fruits and vegetables [10,22,28]. However, it remains unclear whether this dual approach has impacted actual consumption, as no systematic data on population consumption have been collected following the policy adoption in 2012. 

There is also a lack of adequate monitoring and evaluation of the impact of NCD-related fiscal policies in PICs [10,20,24,29,30,31]. Without regular systematic surveillance, it remains unclear whether, how, or to what extent the interventions have impacted the food choices of Pacific Islanders. It is also critical to build an evidence base of the most cost-effective fiscal programs so that decision-makers continue to support health-related policies as aligned with other economic and trade priorities [10,20,31,32]. To date, the evidence in support of tax policies has come in largely anecdotal or indirect form, such as observations of the low-sugar options available to consumers and the higher retail prices of SSBs [10]. Similarly, justifications for the economic value of these policies come from simulations and predictive models indicating the positive association between tax increases and consumption decreases [10,19,20]. Nonetheless, there are examples of investigations into PIC consumer behaviour using qualitative interviews. For example, interviews with consumers and retailers in Fiji reported in two recent studies showed little awareness of the health-taxation price changes at the consumer level [14]. These studies also indicate that the low consumption of fruits vegetables and the high consumption of unhealthy foods may be due to socio-cultural factors, such as attaching negative connotations with eating vegetables whilst valuing meat, having a preference for locally grown fruits and vegetables and having a desire for fast foods and ultra-processed snacks [14,33,34]. These qualitative studies provide important indicators for what needs to be included in a systematic evaluation of consumer behaviour and food choices in Pacific populations. 

Given that fiscal policies are a key component of NCD-related intervention in PICs, it is important that the impact of the taxation on consumer behaviour and population health data is regularly monitored during the taxation period. In order to provide data to evaluate food-related fiscal policies, the WHO STEPwise survey was recommended to strengthen surveillance of NCD policy development across the PICs. However, this systematic surveillance approach has not been undertaken consistently or frequently enough to provide the necessary outcome data [8,30]. For example, it was adopted by the Fiji Ministry of Health in 2001 as part of the long-term strategy for NCDs [35], but there have only been two iterations, in 2002 and 2011, and a proposed 2018 survey was postponed until 2023 [31]. This situation is similar across PICs, with most countries having implemented only one (Niue, Tuvalu) or two surveys at inconsistent time points due to the cost and logistical challenges involved [12]. It is important to have a range of research data on consumption and food-choice attitudes in order to fully understand consumer behaviour and, therefore, guide NCD-related policy.

The present study aims to support this broader research need. It provides data on the consumption of and household access to SSBs, ultra-processed packaged snacks, fruits and vegetables and attitudes of consumers on food prices and unhealthy eating from population samples from six PICs: Fiji, Kiribati, Samoa, Solomon Islands, Tonga, and Vanuatu. The study also assesses the relationship between consumption of SSBs, ultra-processed packaged snacks, fruits and vegetables and (1) household access, (2) price as a food choice motive, and (3) unhealthy eating attitudes. Therefore, this study provides novel data on food-related behaviours in PICs and offers insights into the potential impact of NCD-related fiscal policies on food consumption. 

## 2. Materials and Methods

### 2.1. Study Design and Study Population 

The data used in this study are part of a larger ongoing study on food choice motives and eating behaviour in PICs led by the first author. The present study reports on data collected from April 2021 to November 2022. Data were collected through an online cross-sectional survey. Participants were recruited using convenience sampling. Psychology undergraduate students of the University of the South Pacific (USP) assisted with participant recruitment by sharing the participant information sheet and a survey link with their close contacts. USP is a regional university, owned and governed by 12 member PICs: Cook Islands, Fiji, Kiribati, Marshall Islands, Nauru, Niue, Samoa, Solomon Islands, Tokelau, Tonga, Tuvalu and Vanuatu [36]. Approximately 7210 invitations to participate in the study were sent out, and usable data were collected from 4620 participants (participation rate 64%). Pacific Islanders were eligible for participation in this study if they were over the age for 18 years, fluent in English and gave consent for participation after reading the Participant Information Sheet. Participants from PICs with low numbers of participants were removed from the data file: Cook Islands (*n* = 15), Marshall Islands (*n* = 4), Niue (*n* = 2), Tokelau (*n* = 6), Tuvalu (*n* = 36). Further, participants who did not specify their nationality (*n* = 99) or selected “other” (*n* = 342) were also removed from the data file, as we could not identify the PIC they were living in and the duration of stay, both of which are crucial for assessing exposure to fiscal policies. 

### 2.2. Measures 

#### 2.2.1. Consumption of SSBs, Ultra-Processed Packaged Snacks, Fruits and Vegetables

The daily consumption of glasses of SSBs, units of ultra-processed packaged snacks, and servings of fruits and vegetables was measured using four questions on dietary intake, adapted from De Vet et al. [37]. An example of an items is: “How many glasses of soft drinks (Coke/Fanta/Sprite etc.), lemonade or energy drinks (V drink/Red Bull/Mother etc.) do you drink on an average day? (Don’t count light soft drinks like diet coke and mineral water)”. A six-point scale was used to measure consumption, and response options included “less than 1”, “1”, “2”, “3”, “4”, and “more than four”. The questions on SSBs and ultra-processed packaged snacks were also modified to draw on examples from PICs. 

#### 2.2.2. Access to SSBs, Ultra-Processed Packaged Snacks, Fruits and Vegetables

Access to SSBs, ultra-processed packaged snacks, fruits and vegetables was measured using three questions on the frequency of access to the type of food or drink at home, for example, “Do you have access to carbonated drinks (like Coke, Fanta, Pepsi), lemonade or energy drinks (e.g., V drink/Red Bull/Mother etc.) (Don’t count: mineral water or light sodas, such as diet coke) at home?” Responses could be indicated on a six-point scale, with response options “never”, “rarely”, “sometimes”, “often”, and “always” used.

#### 2.2.3. Price Considerations

Three items from the Food Choice Questionnaire [38] were included to measure price as a food choice motive. Participants responded to the prompt “It is important to me that the food I eat on a typical day”, which was followed by the three items: “is not expensive”, “is good value for money” and “is cheap”. A four-point response scale was used, with options “not important at all”, “a little important”, “moderately important” and “very important”. The scale had acceptable internal reliability (Cronbach’s alpha = 0.67).

#### 2.2.4. Self-Reported Body Mass Index (BMI)

Participants were asked to give their best estimates for their height and weight in metric units; any measurements given in imperial units were converted. Each participant’s body mass index (BMI) was calculated by dividing weight in kilograms by height in meters squared. BMI was categorized for descriptive analyses based on WHO recommendations: BMI less than 18.5—underweight range, BMI 18.5 to 24.9—healthy weight range, BMI 25.0 to 29.9—overweight range, and BMI 30.0 or higher—obese range [39]. All participants reporting implausible BMI values (*n* = 41) lower than 16 or higher than 40 were also removed from the sample [39].

#### 2.2.5. Unhealthy Eating Attitudes

In an earlier qualitative study, we found reference to the notion of “kana meda bula”, which translates into “eat to live” and to enjoy the food and the company [33]. The phrase is commonly used in social gatherings to encourage overeating and unhealthy eating by encouraging “letting go of any inhibitions that there might be, such as concerns for health or weight” [33] (p. 5). Therefore, a simple measure of unhealthy eating attitudes was included here with the single item “You only live once so I eat foods that I love even if they are not very healthy”. A five-point scale was used to assess response, with options including “strongly agree”, “agree”, “disagree”, and “strongly disagree”.

### 2.3. Statistical Analysis

IBM SPSS Statistics for Windows (version 29) was used for data analysis. Data are presented as percentages to allow easier comparison between the countries with different sample sizes. The Chi square test of independence was used to test for statistical differences by country. Cramer’s V was used to test for strength of association. Effect sizes for Cramer’s V were interpreted using Cohen’s [40] conventions based on the number of levels for the variable with lowest levels. One-way analysis of variance (ANOVA) was used to test for statistically significant differences in price by country; the equality of variances requirement for ANOVA was met. Since the data did not meet the assumptions of ordinal logistic regression, a binary logistic regression analysis was conducted to examine the relationship between the consumption of SSBs, ultra-processed packaged snacks, fruits and vegetables and (1) nationality, (2) household access to these foods and drinks, (3) price of food as a choice motive and (4) unhealthy eating attitudes. The consumption variables were recoded into dichotomous variables (0 = less than 1 glass/unit/serving, 1 = one or more glasses/units/servings). The omnibus tests of model coefficients and the Hosmer–Lemeshow test were assessed to appraise model fit. An additional hierarchical logistic regression analysis using the Baron–Kenny [41] method for mediation was conducted to assess a potential impact of price on consumption via household access. The level of significance was set at 0.05.

### 2.4. Ethical Approval

Ethical approval for the study was given by the Research and Innovation Office of USP. Participants received detailed information regarding the purpose of the study and their rights regarding participation and withdrawal, and were assured of complete anonymity. All participants provided informed consent before the start of the study.

## 3. Results 

### 3.1. Sample Characteristics

The sample consisted of 4116 participants who were predominantly Fijian (60.7%), urban (82.85%), female (66.7%) and highly educated (at least 70% had a university qualification). Mean age was 30.04 years (sd = 10.34), and 88.9% of the sample provided usable data to calculate BMI. Of these, 4.6% were underweight, 32.9% had normal weight, 26% were overweight and 36.6% were obese. The median BMI for the sample was 27.04. Table 1 shows participant characteristics by country. 

### 3.2. Consumption and Access to Types of Food and Drink, by Country

Participants’ reported consumption of SSBs and ultra-processed packaged snacks, fruits and vegetables by country is shown in Table 2. Overall, the majority of respondents self-reported daily consumption of one or less than one glass of SSBs (69.4%), two or fewer ultra-processed packaged snacks (58.8%) and two or fewer servings of fruits (79.4%) and vegetables (60%). Small-to-medium statistically significant differences in consumption and access were found across the six PICs for SSBs, ultra-processed packaged snacks, fruits and vegetables. Ni-Vanuatu participants reported the lowest consumption rates of SSBs, and Tonga the highest. Fiji and Vanuatu had the lowest rates of ultra-processed packaged snack consumption, while Tonga and Samoa reported the highest daily consumption. Solomon Islands had the highest rates of fruit and vegetable consumption, Samoa and Fiji the lowest fruit consumption, and Kiribati had the lowest vegetable consumption rates. According to Cohen’s guidance [40], these associations between nationality and consumption of foods and drink can be characterized as “small”.

Table 3 shows the participants’ reports of household access to SSBs, ultra-processed packaged snacks, fruits and vegetables, by country. Fiji and Samoa had the highest household access to SSBs, and Tonga and Vanuatu had the lowest. Fiji and Samoa also had the highest household access to ultra-processed packaged snacks, whereas Solomon Islands had the lowest. Fiji and Vanuatu had the highest household access to fruits and vegetables, and Kiribati had the lowest. Overall, participants had higher household access to fruits and vegetables than SSBs and ultra-processed packaged snacks. The associations between nationality and access to foods and drink can be characterized as “small-to-medium” [40]. 

### 3.3. Importance of Price as a Food Choice Motive, by Country 

Participants across all countries indicated price as a motive for food choices as moderately to very important. The importance of price as a motive for choosing food differed across the six countries, *F* (5, 4057) = 10.7, *η*^2^ = 0.13, *p* = <0.01. However, the differences could be characterised as small [40]. Fijian and i-Kiribati participants placed the most emphasis, and Ni-Vanuatu the least emphasis, on price while choosing food. Table 4 presents the mean and standard deviation by country.

### 3.4. Attitudes toward Unhealthy Eating, by Country 

Table 5 shows the relative level of agreement with each of the attitude statements across the six PICs. Small but statistically significant associations were identified across the six countries. Overall, slightly more respondents agreed with eating irrespective of health consequences. 

### 3.5. Relationship between Consumption of Different Types of Food and Drink and Price as a Food Choice Motive, Household Access and Unhealthy Eating Attitudes

A binary logistic regression analysis was conducted to examine the relationship between the consumption of SSBs, ultra-processed packaged snacks, fruits and vegetables and four predictor variables: nationality, household access to the food or drink, price as a food choice motive, and unhealthy eating attitudes. Few significant country differences were found in the consumption of SBBs, fruits and vegetables. Findings do show that consumption of ultra-processed packaged snacks was significantly higher in Kiribati than in most other countries (only the difference with Tonga was non-significant). Household access and unhealthy eating attitudes were the strongest predictors of increased consumption of SSBs and ultra-processed packaged snacks. Household access was also the strongest predictor of increased fruit and vegetable consumption, whereas unhealthy attitudes decreased fruit and vegetable consumption. The relationships between price as food choice motive and fruit consumption were not statistically significant. Table 6 presents the results. 

Since fiscal policies are aimed at reducing access by increasing price, a follow-up hierarchical regression using the Baron–Kenny [41] method for mediation was conducted to assess if price as food choice motive was associated with household access, which in turn impacted consumption of foods and drink. To establish this mediation, price as food choice motive was entered into the hierarchical logistical analyses first, followed by household access. The associations between price as food choice motive and consumption of SSBs, ultra-processed packaged snacks, fruits and vegetables became stronger after adding household access, indicating no mediation effect. Mediation would have been indicated by associations between price as food choice motive and consumption becoming non-significant or smaller. The results are presented in Appendix A. 

## 4. Discussion 

This study presented data on daily consumption and household access to SSBs, ultra-processed packaged snacks, fruits and vegetables and attitudes towards price food choice and unhealthy eating from six PICs (Fiji, Kiribati, Samoa, Solomon Islands, Tonga and Vanuatu). Additionally, this study assessed the potential impact of NCD-related fiscal policies on food consumption by examining the relationship between consumption of these foods and drink and (1) household access, (2) price as food choice motive, and (3) unhealthy eating attitudes. The findings of the study indicate that consumption of fruits and vegetables among participants from the countries studied continues to be low; 80–90% of the participants still did not meet the guidelines for daily consumption of fruits and vegetables, showing no substantial change from earlier reports [11,12]. This finding also suggests that dietary interventions to increase consumption of fruits and vegetables in these countries may not have been impactful within this population in the past decade. For example, the removal of tax on imported fruits and vegetables in Fiji in 2014 has not been reflected in an overall increase in the consumption of fruits and vegetables as it was intended to achieve. Our data indicate that 81% of Fijians eat two or less servings of fruits, and 58% eat two or less servings of vegetables in a day. Taken together, these findings suggest that the majority of Fijians are likely to consume less than the daily recommended five or more servings of fruits and vegetables. Furthermore, the median BMI across the sample was well within the overweight range, and over one-third of respondents in this study reported height and weight estimates that placed them in the obese range. This further confirms the limited impact of single policy interventions on reducing obesity at the population level, and underscores the need for a systems approach to reducing obesity and overweight within this population. 

A positive finding was that in comparison to earlier studies in PICs [12], SSB consumption was relatively lower across the six countries, with 40–60% participants reporting consuming less than one glass of SSB in a day and only 13% of the participants reporting high levels of SSB consumption (i.e., three or more glasses of SSBs daily), similarly to previous studies [12]. The changes in SSB-related taxation in the six countries may offer some insights into how fiscal policies may impact consumption. Fiji and Vanuatu reported the lowest SSB consumption, with 48% and 38% participants reporting consuming more than one glass of SSB daily, respectively. Both of these countries have increased taxes on SSBs by 20% since 2000 [22]. On the other hand, Kiribati and Solomon Islands reported the highest daily consumption of SSBs, with approximately 62% of participants from both countries reporting consuming one or more glasses of SSBs daily. SSB-related taxes have decreased by approximately 20% in both of these countries since 2000 [22]. 

However, Samoa, where an SSB-related tax has remained the same since 2000, also reported comparatively lower daily consumption of SSBs (53%) than Kiribati and Solomon Islands. Tonga was the only country in this sample which increased taxes on imported SSBs whilst decreasing taxes on locally produced SSBs [22], and approximately 58% of Tongan participants reported consuming one or more glasses of SSBs daily. Tonga also reported one of the highest daily SSB consumption levels in this sample: 23% of Tongan participants consumed three or more glasses of SSBs daily. Teng et al. [22] suggest that the comparatively lower taxes on locally produced SSBs than imported SSBs can be counterproductive, as consumers may opt for cheaper options. 

The average tax on SSBs and tax per litre of SSB (in USD) data compiled by Teng et al. [22] also seem inconsequential for SSB consumption in this sample. For instance, in comparison to the other countries in the sample, Vanuatu had the highest SSB-related taxes and highest tax per litre and the lowest consumption. However, Samoa, which has one of the lowest taxes in the sample and the lowest tax per litre of SSB, had lower SSB consumption than countries like Tonga and Kiribati, which have higher average taxes on SSBs and higher taxes per litre of SSB. Taken together, these inconsistencies indicate the lack of any clear patterns in average SSB taxes and tax per litre of SSBs and the corresponding levels of SSB consumption. These findings further reinforce that (1) SSB-related tax design flaws (e.g., having lower taxes on locally produced SSBs) and tax reduction may impede the impacts of SSB-related fiscal policies and (2) other factors (in addition to price) also impact consumption. 

Teng et al. [22] also suggest that the tax design is an important consideration in relation to the effectiveness of SSB-related fiscal policies. The tiered volumetric design used by Tonga, which applies taxes directly to sugar content, is rated as the most effective as it discourages a shift to cheaper options, followed by the volumetric tax design used by Fiji, Samoa and Vanuatu, which taxes on the volume of SSBs. Finally, the ad valorem tax design as used by Kiribati is least effective, as it taxes on the value of the SSB rather than the sugar content, allowing easy change to cheaper alternatives [22]. However, since Fiji and Tonga have lower taxes on locally produced SSBs, SSB taxation in these countries may be largely ineffective, irrespective of the tax design used, as this also allows switching to cheaper alternatives [22]. Our data suggest that the relative change (increase or decrease) in SSB prices due to taxation may have a greater impact on SSB consumption than the percentage of tax, tax per litre or tax design used, especially if cheaper SSB alternatives are available.

A notable finding of this study was that the consumption of servings of ultra-processed packaged snacks was higher than consumption of servings of SSBs: 66% of participants ate one or more packets of ultra-processed packaged snacks daily. The higher consumption of ultra-processed packaged snacks may in part be explained by the fact that NCD-related fiscal policies in the PICs have predominantly focused on reducing sugar intake from SSB import and sales, whilst fewer policies exist for other unhealthy foods such as ultra- processed snacks [22,24]. While the current data suggest that there may have been an impact of this comparative difference in policy targets, inter-country comparisons on consumption of unhealthy foods and drinks reveal interesting results. Similarly to SSB consumption, consumption of ultra-processed packaged snacks was higher amongst i-Kiribati, Samoan, Solomon Islands and Tongan participants and lower amongst Fijian and Ni-Vanuatu participants. The inter-country similarities in unhealthy eating practices in consumption of SSBs and ultra-processed packaged snacks point to factors other than price that might also impact consumption of these foods. For instance, in earlier qualitative studies, Buksh et al. [33,34] recorded an affinity towards ultra-processed snacks amongst Fijian participants, suggesting that taste was an important factor impacting consumption. Furthermore, Samoa and Tonga were the only countries in this sample with fiscal policies targeting sugary snacks. However, both countries reported high consumptions of ultra-processed packaged snacks, with Tongan participants reporting the highest consumption levels in the sample, indicating that fiscal policies on sugary snacks may have limited impact on the consumption of ultra-processed snacks in these countries. 

To further the understanding of the potential impacts of fiscal policies (through changes in price) on the consumption of healthy and unhealthy foods and to examine other variables that may impact consumption, this study used regression analyses to examine the relationship between the consumption of SSBs, ultra-processed packaged snacks, fruits and vegetables and four predictor variables: nationality, household access to the food or drink, price as food choice motive, and unhealthy eating attitudes In combination, the four predictor variables could explain 20% and 16%, respectively, of SSB and snack consumption, and 6.5% and 13%, respectively, of fruits and vegetable respectively. Household access and unhealthy eating attitudes were the strongest predictors of increased consumption of SSBs and ultra-processed packaged snacks. Household access was also the greatest predictor of fruit and vegetable consumption, whereas unhealthy attitudes decreased fruit consumption and were not related to vegetable consumption. Furthermore, follow-up analyses ruled out a mediation impact of price on household access to foods and drink.

Our findings indicate that while food choices may be impacted by price to some extent, the drivers of the consumption of foods and drinks are likely more complex than presumed by fiscal policy modelling exercises, e.g., [18,19,20], and require further exploration. Specifically, our regression analyses support the findings from qualitative studies that low consumption of fruits and vegetables and the consumption of SSBs and ultra-processed packaged snacks are not simply driven by price of foods and drink but are also closely tied to sociocultural attitudes and preferences [10,33,42,43]. Works in the literature from PICs indicate food as central to sustaining social relationships within the PIC communal structures [33,42,43], where eating should be without inhibitions around health or weight [33], a larger ‘well-nourished’ body is a sign of positive social standing [33,42], health and happiness [33], and unhealthy foods known to be valued and loved by others are purchased irrespective of health outcomes [33]. Therefore, in countries where SSB-related fiscal policies are not used effectively (e.g., due to tax design flaws, having lower taxes on locally produced SSBs), consumers may opt for cheaper alternatives to satisfy the appetite for SSBs and ultra-processed snacks. Similarly, reducing taxes may also incentivize sales of valued unhealthy foods such as SSBs and ultra-processed snacks. 

The finding of low fruit and vegetable consumption despite access, and of high BMI despite reportedly low unhealthy food consumption, also may suggest a lack of diet and nutrition awareness in PIC populations. That is, Pacific Islanders may perceive fruits and vegetables as healthy but have poor understanding of the quantity that have to be consumed [44]. Buksh et al. [33] found that Fijians talked about vegetables as being of low social value and not indicative of a good meal, and that consumption of calorie-dense foods was high because they were viewed as more satiating. Similarly, Pacifica mothers described switching from SSBs to equally unhealthy fruit concentrates, as the latter were perceived as being healthier [44]. Additionally, PIC populations may not be aware that the levels of sugar and salt in single servings of SSBs and ultra-processed snacks are beyond daily limits, or what a standard serving is compared to the size of the purchased item (e.g., size of SSB bottle or box of snacks). To some extent, the fiscal strategies may have assumed that populations have an innate knowledge of healthy diet and nutrition but are choosing otherwise when unhealthy, high sugar foods are easily accessible. However, these findings underscore the need for investment in nutrition education interventions that target attitudes and values that promote unhealthy eating in PICs. Therefore, it is important to include measures of attitudes towards healthy and unhealthy food choices as part of any evaluation of NCD-related strategies and to consider utilizing the revenue from NCD-related fiscal policies for public attitude and behaviour change campaigns [16,22,33,34,38]. 

### Strengths and Limitations 

This study was the first of its kind to explore the impact of the widely promoted fiscal policies on the consumption of SSBs in PICs through an examination of population data. The findings highlight potential limitations of the use of fiscal policies and identify additional variables of interest which may impact consumption: household access and unhealthy eating attitudes. However, this study has several limitations that need to be considered when interpreting the results. This study used convenience sampling methods, resulting in a sample that was predominantly urban and educated, which increased both participants’ physical access to SSBs and ultra-processed packaged snacks as well as the affordability of these foods. Nonetheless, the sample is still relevant, as higher rates of obesity have been recorded in urban samples in PICs [9]. Furthermore, while the country sub-samples were based largely on USP student enrolment in these countries, they also represented the population size in these countries, with Fiji having the largest population of the six countries (Appendix B) [45]. Moreover, this study used only household access as an indicator of access to ultra-processed foods and SSBs, whilst PIC data indicate that purchasing these through small retail outlets (e.g., corner shops and canteens) is common amongst Pacific Islanders [10,46]. Therefore, assessing purchases of SSBs and ultra-processed snacks outside the home (e.g., during transit) is a critical additional indicator of access to unhealthy foods and drinks in the Pacific. Also, only one item was used to assess unhealthy eating attitudes, and future studies will benefit from including multiple items to validly measure unhealthy eating attitudes. In addition, due to the cross-sectional study design, no claims of causality between patterns of consumption of SSBs and SSB-related fiscal policies in the six countries can be made. Lastly, this study represents a preliminary investigation of the drivers of consumption of ultra-processed packaged snacks and SSBs in PICs. Further, more extensive research is needed to gain a deeper understanding of the diversity of drivers of unhealthy food consumption and overweight in PICs. For instance, there is little research on the role of marketing and advertising, the impact of sedentary behaviours such as extended periods of television watching or phone use, and the interplay between emotional states and consumption of unhealthy foods and drinks in PICs. An understanding of these and other potential drivers is needed to develop effective policies and interventions.

## 5. Conclusions

The sustained low consumption of fruits and vegetables and the high rates of overweight and obesity in this sample indicate that NCD-related policies have had limited impact in increasing healthy eating behaviours and reducing weight gain in this population. Furthermore, our findings indicate that the relationship between price and consumption is not as straightforward as NCD-related fiscal strategies have perhaps assumed. The findings also highlight design flaws in the application of SSB-related fiscal policies that impede their success in the six studied PICs. This study adds to the growing body of literature indicating that diet-related NCD interventions targeting PIC populations need to take into account the cultural norms, values and beliefs around food choices and eating behaviours [10,33,42,43,44]. The study findings also highlight the importance of including measures of attitudes towards healthy and unhealthy food choices and behaviours as part of any national survey that expects to inform NCD-related interventions and to consider utilizing the revenue from NCD-related fiscal policies for public attitude and behaviour change campaigns [16,22]. While this preliminary investigation highlights some factors that impact the consumption of unhealthy foods and drinks in PICs, more extensive research is required for a deeper understanding of the drivers of unhealthy eating and the barriers to healthy eating in PICs in order to develop effective policies and interventions. 

## Figures and Tables

**Table 1:** Participant characteristics by country.

	Fiji *n* = 2500	Kiribati *n* = 304	Samoa *n* = 99	Solomon Islands *n* = 753	Tonga *n* = 130	Vanuatu *n* = 317	Total *N* = 4116	*p*
**Age** mean (SD)	30.39 (10.87)	28.92 (10.52)	29.38 (10.98)	28.88 (9.13)	30.40 (8.38)	31.15 (8.69)	30.04 (10.34)	<0.001
**Sex**								<0.001
Female	1716 (68.6%)	226 (74.3)	71 (71.7)	430 (56.9)	94 (72.3)	201 (63.4)	2738 (66.7)	
Male	784 (31.4%)	78 (25.7)	28 (28.3)	323 (43.1)	36 (27.7)	116 (36.6)	1368 (33.3)	
**Residence**								<0.001
Urban	2071 (82.7)	262 (86.2)	85 (85.9)	654 (86.3)	100 (76.9)	233 (74.0)	3405 (82.8)	
Rural	433 (17.3)	42 (13.8)	14 (14.1)	107 (13.7)	30 (23.1)	82 (26.0)	705 (17.2)	
**Education**								<0.001
Primary school or lower	44 (1.8)	0 (0.0)	2 (2.0)	14 (1.8)	0 (0.0)	4 (1.3)	64 (1.6)	
high school or secondary	586 (23.5)	60 (19.7)	22 (22.2)	114 (15.0)	24 (18.6)	84 (26.5)	890 (21.7)	
Vocational training	133 (5.3)	36 (11.8)	8 (8.1)	42 (5.5%)	19 (14.7)	38 (12.0)	276 (6.7)	
University	1731 (69.4)	208 (68.4)	67 (67.7)	588 (77.6)	86 (66.7)	191 (60.3)	2871 (70)	
**Number of people in household**								<0.001
1–5	1727 (69.6)	146 (47.7)	68 (68.7)	411 (54.7)	50 (38.5)	198 (62.9)	2600 (63.7)	
6–10	667 (26.9)	132 (43.1)	27 (27.3)	272 (36.2)	80 (61.5)	109 (34.6)	1287 (31.5)	
11 or more	88 (3.5)	28 (9.2)	4 (4.0)	69 (9.2)	0 (0.0)	8 (2.5)	197 (4.8)	
**BMI**	*n* = 2289	*n* = 271	*n* = 96	*n* = 614	*n* = 118	*n* = 272	*n* = 3660	<0.001
Underweight	110 (4.8)	0 (0.0)	2 (2.1)	41 (6.7)	2 (1.7)	13 (4.8)	168 (4.6)	
Normal Weight	806 (35.2)	69 (25.5)	14 (14.6)	208 (33.9)	26 (22.0)	80 (29.4)	1203 (32.9)	
Overweight	593 (25.9)	71 (26.2)	26 (27.1)	161 (26.2)	21 (17.8)	79 (29.0)	951 (26)	
Obese	780 (34.1)	131 (48.3)	54 (56.3)	204 (33.2)	69 (58.5)	100 (36.8)	1338 (36.6)	
Median	26.56	29.07	31.12	26.34	33.39	27.56	27.04	

Note: Chi square test was used to determine significant differences between groups.

**Table 2:** Daily consumption of SSBs, ultra-processed packaged snacks, fruits and vegetables, by country.

	Fiji	Kiribati	Samoa	Solomon Islands	Tonga	Vanuatu	Total	Cramer’s V	*p*
% participants									
**SSBs**								0.091	<0.001
less than 1	52.4	37.9	46.5	38.5	41.5	61.6	49.0		
1	17.8	24.2	9.1	27.3	23.8	23.2	20.4		
2	16.2	21.6	28.3	21.8	11.5	10.8	17.3		
3	7.4	12.4	6.1	9.0	14.6	2.5	7.9		
4	2.2	2.0	2.0	0.8	3.8	1.6	1.9		
more than 4	4.1	2.0	8.1	2.7	4.6	0.3	3.5		
**Ultra-processed packaged snacks**								0.062	<0.001
less than 1	36.7	20.9	29.3	32.5	22.3	35.9	34.1		
1	24.8	24.8	26.3	24.1	21.5	26.3	24.7		
2	21.7	31.4	20.2	24.1	27.7	23.8	23.2		
3	10.7	16.3	10.1	12.3	17.7	9.8	11.5		
4	2.6	1.3	4.0	2.9	4.6	2.5	2.7		
more than 4	3.5	5.2	10.1	4.0	6.2	1.6	3.8		
**Fruits**								0.075	<0.001
less than 1	22.6	19.1	22.2	16.4	19.2	17.7	20.7		
1	31.3	27.6	34.3	22.5	34.6	28.4	29.4		
2	27.2	35.5	25.3	32.1	23.1	37.2	29.3		
3	12.6	15.1	10.1	17.4	10.8	9.1	13.3		
4	2.5	1.3	8.1	5.3	6.2	3.8	3.3		
more than 4	3.7	1.3	0.0	6.2	6.2	3.8	4.0		
**Vegetables**								0.078	<0.001
less than 1	9.4	19.6	14.1	10.0	13.1	4.7	10.1		
1	18.2	24.2	20.2	18.1	15.4	20.2	18.7		
2	30.7	30.1	28.3	29.3	37.7	39.4	31.2		
3	21.5	16.3	15.2	26.7	16.2	18.9	21.5		
4	7.4	2.6	10.1	9.6	11.5	7.6	7.7		
more than 4	12.9	7.2	12.1	6.3	6.2	9.1	10.7		

Note: A serving of SSB is defined as one glass. A unit of ultra-processed packaged snacks is defined as a small packet of salty snack or a small bar of chocolate. One serving of fruits and vegetables is defined as approximately one handful. Chi square test was used to determine significant differences between groups. Cramer’s V was used to test for strength of associations.

**Table 3:** Household access to SSBs, ultra-processed packaged snacks, fruits and vegetables, by country.

	Fiji	Kiribati	Samoa	Solomon Islands	Tonga	Vanuatu	Total	Cramer’s V	*p* Value
% participants									
**SSBs**								0.08	<0.001
never	11.2	7.9	12.1	14.6	6.9	15.8	11.8		
rarely	26.7	29.6	20.2	20.3	30.8	31.9	26.1		
sometimes	36.0	42.8	31.3	46.8	47.7	39.4	39.0		
often	14.9	15.1	24.2	9.9	6.9	7.6	13.4		
always	11.2	4.6	12.1	8.4	7.7	5.4	9.7		
**Ultra-processed packaged snacks**								0.10	<0.001
never	5.4	5.9	0.0	11.1	3.1	6.3	6.4		
rarely	19.3	18.3	14.1	17.2	16.9	21.0	18.7		
sometimes	37.2	50.3	43.4	50.5	55.4	45.7	42.0		
often	19.7	17.6	24.2	12.9	13.1	20.0	18.2		
always	18.5	7.8	18.2	8.3	11.5	7.0	14.7		
**Fruits and vegetables**								0.15	<0.001
never	1.8	3.8	0.0	1.1	1.5	0.0	1.6		
rarely	3.6	13.9	18.2	3.3	3.1	2.5	4.6		
sometimes	23.9	52.0	26.3	40.5	41.5	32.8	30.3		
often	26.2	19.1	20.2	16.7	27.7	19.2	23.3		
always	44.5	11.2	35.4	38.4	26.2	45.4	40.2		

Note: Chi square test was used to determine significant differences between countries. Cramer’s V was used to test for strength of associations.

**Table 4:** Participants’ ratings of importance of price for food choice, by country.

	*n*	Mean (SD)	Median
Fiji	2481	3.18 (0.70)	3.33
Kiribati	306	3.18 (0.69)	3.33
Samoa	95	3.16 (0.66)	3.00
Solomon Islands	744	3.03 (0.71)	3.00
Tonga	126	3.17 (0.66)	3.33
Vanuatu	311	2.93 (0.79)	3.33
Total	4063	3.13 (0.71)	3.33

**Table 5:** Unhealthy eating attitudes, by country.

	Fiji	Kiribati	Samoa	Solomon Islands	Tonga	Vanuatu	Total	Cramer’s V	*p* Value
% participants									
**You only live once so I eat foods that I love even if they are not very healthy**								0.11	<0.001
Strongly agree	16.2	21.7	10.1	11.9	16.2	9.8	15.2		
Agree	42.1	50.7	49.5	31.4	36.2	29.0	39.7		
Disagree	30.1	23.7	34.3	41.6	34.6	38.5	32.7		
Strongly disagree	11.6	3.9	6.1	15.0	13.1	22.7	12.4		

The chi-square test was used to determine differences across the six countries, and Cramer’s V was used to measure strength of the differences.

**Table 6:** Relationship between consumption and nationality, household access, price as food choice motive and unhealthy eating attitudes.

	B	SE	Wald	*p*	OR	95% CI
**SSB** Nagelkerke R Square = 0.202						
Nationality						
Kiribati	Reference					
Fiji	−0.573	0.133	18.697	<0.001	0.564	−0.833–−0.313
Samoa	−0.392	0.255	2.353	0.125	0.676	−0.892–0.109
Solomon Islands	0.254	0.150	2.856	0.091	1.289	−0.041–0.549
Tonga	0.116	0.231	0.252	0.616	1.123	−0.337–0.568
Vanuatu	−0.615	0.178	11.884	<0.001	0.541	−0.965–−0.265
Household access	0.597	0.034	306.803	<0.001	1.817	0.530–0.664
Price as food choice motive	0.131	0.049	7.176	0.007	1.139	0.035–0.266
Unhealthy eating attitude	0.460	0.041	127.309	<0.001	1.584	0.38–0.54
**Ultra-processed packaged snacks** Nagelkerke R Square = 0.162						
Nationality						
Kiribati	Reference					
Fiji	−0.839	0.154	29.802	<0.001	0.432	−1.140–−0.538
Samoa	−0.664	0.277	5.769	0.016	0.515	−1.206–−0.122
Solomon Islands	−0.335	0.169	3.910	0.048	0.716	−0.666–−0.003
Tonga	0.063	0.269	0.056	0.814	1.065	−0.463–0.590
Vanuatu	−0.423	0.195	4.712	0.030	0.655	−0.804–−0.041
Household access	0.544	0.036	234.320	<0.001	1.723	1.671–2.691
Price as food choice motive	0.230	0.050	21.266	<0.001	1.259	0.132–0.328
Unhealthy eating attitude	0.429	0.042	106.736	<0.001	1.536	0.348–0.510
**Fruits** Nagelkerke R Square = 0.065						
Nationality						
Kiribati	Reference					
Fiji	−0.615	0.160	14.717	<0.001	0.541	−0.930–−0.301
Samoa	−0.503	0.291	2.993	0.084	0.605	−1.073–0.067
Solomon Islands	−0.180	0.182	0.981	0.322	0.835	−0.537–0.177
Tonga	−0.319	0.273	1.372	0.242	0.727	−0.854–0.215
Vanuatu	−0.374	0.215	3.012	0.083	0.688	−0.796–0.048
Household access	0.433	0.039	121.995	<0.001	1.542	0.356–0.510
Price as food choice motive	0.080	0.056	2.021	0.155	1.083	−0.030–0.189
Unhealthy eating attitude	−0.207	0.046	20.246	<0.001	0.813	−0.298–−0.117
**Vegetables** Nagelkerke R Square = 0.139						
Nationality						
Kiribati	Reference					
Fiji	0.249	0.172	2.082	0.149	1.281	−0.089–0.587
Samoa	−0.013	0.341	0.001	0.970	0.987	−0.681–0.655
Solomon Islands	0.381	0.203	3.521	0.061	1.464	−0.017–0.780
Tonga	0.171	0.323	0.280	0.597	2.909	−0.463–0.804
Vanuatu	1.068	0.313	11.605	<0.001	1.186	0.453–1.682
Household access	0.770	0.054	203.712	<0.001	0.969	0.665–0.876
Price as food choice motive	0.297	0.075	15.536	<0.001	1.346	0.149–0.445
Unhealthy eating attitude	−0.032	0.064	0.244	0.622	2.161	−0.157–0.094

## Data Availability

The data presented in this study are not publicly available, as they are part of an ongoing study.

## Appendix A

	**Nagelkerke R Square**	**B**	**SE**	**Wald**	***p***	**OR**	**95% CI**
**SSB**							
**Step 1**	0.001						
Price food choice		0.079	0.044	3.233	0.072	1.083	0.993–1.181
**Step 2**	0.136						
Price food choice		0.138	0.047	8.607	0.003	1.148	1.047–1.259
House hold access		0.637	0.033	375.207	<0.001	1.891	1.773–2.017
**Ultra-processed Snacks**							
**Step 1**	0.004						
Price food choice		0.167	0.046	13.068	<0.001	1.182	1.080–1.294
**Step 2**	0.106						
Price food choice		0.229	0.048	22.546	<0.001	1.258	1.144–1.383
House hold access		0.570	0.034	277.005	<0.001	1.769	1.654–1.892
**Fruits**							
**Step 1**	0.000						
Price food choice		0.044	0.054	0.670	0.413	1.045	0.940–1.163
**Step 2**	0.046						
Price food choice		0.038	0.055	0.487	0.485	1.039	0.933–1.158
House hold access		0.412	0.038	118.798	<0.001	1.510	1.402–1.626
**Vegetables**							
**Step 1**	0.007						
Price food choice		0.265	0.071	14.017	<0.001	1.303	1.134–1.497
**Step 2**	0.130						
Price food choice		0.787	0.052	225.181	<0.001	2.198	1.136–1.516
House hold access		−1.504	0.290	26.952	<0.001	0.222	2.055–2.545
